# Mucosal-associated invariant T cells in patients with axial spondyloarthritis

**DOI:** 10.3389/fimmu.2023.1128270

**Published:** 2023-03-10

**Authors:** Rienk Gerben van der Meer, Anneke Spoorenberg, Elisabeth Brouwer, Berber Doornbos-van der Meer, Annemieke M. H. Boots, Suzanne Arends, Wayel H. Abdulahad

**Affiliations:** ^1^ Department of Rheumatology and Clinical Immunology, University Medical Center Groningen, University of Groningen, Groningen, Netherlands; ^2^ Department of Pathology and Medical Biology, University Medical Center Groningen, University of Groningen, Groningen, Netherlands

**Keywords:** axial spondyloarthritis, immunology, MAIT cells, Granzyme A, pathogenesis

## Abstract

**Background:**

Several studies implicate Th17-cells and its cytokine (IL-17) in disease pathogenesis of spondyloarthritis (SpA), with available evidence supporting a pathogenic role of CD8+ T-cells. However, data on the involvement of CD8+ mucosal-associated invariant T-cells (MAIT) and their phenotypic characterization and inflammatory function including IL-17 and Granzyme A production in a homogenous population of SpA-patients with primarily axial disease (axSpA) are lacking.

**Objectives:**

Quantify and characterize the phenotype and function of circulating CD8+MAIT-cells in axSpA-patients with primarily axial disease.

**Methods:**

Blood samples were obtained from 41 axSpA-patients and 30 age- and sex-matched healthy controls (HC). Numbers and percentages of MAIT-cells (defined as CD3^+^CD8^+^CD161^high^TCR_Vα7.2_^+^) were determined, and production of IL-17 and Granzyme A (GrzA) by MAIT-cells were examined by flow cytometry upon *in vitro* stimulation. Serum IgG specific for CMV was measured by ELISA.

**Results:**

No significant differences in numbers and percentages of circulating MAIT-cells were found between axSpA-patients and HCr zijn meer resultaten de centrale memory CD8 T cellen. cellen van patirculating MAIT cells.. Further phenotypic analysis revealed a significant decrease in numbers of central memory MAIT-cells of axSpA-patients compared to HC. The decrease in central memory MAIT-cells in axSpA patients was not attributed to an alteration in CD8 T-cell numbers, but correlated inversely with serum CMV-IgG titers. Production of IL-17 by MAIT-cells was comparable between axSpA-patients and HC, whereas a significant decrease in the production of GrzA by MAIT-cells from axSpA-patients was observed.

**Conclusions:**

The decrease in cytotoxic capability of circulating MAIT-cells in axSpA-patients might implicate that these cell types migrate to the inflamed tissue and therefore associate with the axial disease pathogenesis.

## Introduction

Spondyloarthritis (SpA) is a group of closely related chronic, auto-inflammatory rheumatic disorders with overlapping clinical features and pathogenic mechanisms. The different diagnoses can be divided into pre-dominantly axial involvement; such as ankylosing spondylitis (AS), and non-radiographic axial SpA or pre-dominantly peripheral involvement; for example psoriatic arthritis (PsA) (1, 2). They can share several clinical features including sacroiliitis and spondylitis and/or peripheral arthritis, enthesitis and dactylitis (3). In addition, extra-skeletal manifestations such as inflammatory bowel disease, psoriasis and uveitis may be present (4).

The pathogenesis of SpA is multifactorial and remains not fully understood. Genetic factors as well as cellular immunity seem to play an important role in disease pathogenesis. The allele HLA-B27 of MHC class I has been identified as the main genetic risk factor for the development of SpA, especially for AS (5, 6). In addition, CD4+ T helper (Th) cells seem to play a crucial role in the development of SpA (7). It is hypothesized that HLA-B27 is promoting T-cell survival through binding of killer cell immunoglobulin-like receptor 3 DL2 (KIR3DL2) to HLA-B27 on Th cell surface (8, 9). Furthermore, these KIR3DL2+ CD4 Th cells produce interleukin-17 (IL-17) and carry markers attributed to the Th17 phenotype, like CCR6 and CD161 (9). IL-17, together with IL-23 have been identified as important factors in the immunopathogenesis of peripheral arthritis in SpA (10, 11). Subclinical gut inflammation and dysbiosis of microbiota in the gut are hypothesized to drive the immune response with the production of IL-23 by the gut epithelium and IL-17 by intestinal lymphocytes (11). IL-17 has also been found increased in serum and synovial fluid of SpA patients with peripheral arthritis (12–15). In another line of studies, researchers have proposed a major role of CD8 T cells and NK cells in the pathogenesis of AS due to changes in their cytotoxic profile. Data by Gracey and co-workers suggested that CD8 T cells with a cytotoxic phenotype are recruited to the joints and may play an overlooked role in the pathogenesis of AS (16, 17). In addition, recent study by Ren et al. uncovered remarkable differences in NK cell subsets and cytotoxic profile between AS patients and healthy individuals (18). Importantly, they have shown that plasma levels of Granzyme A (GrzA) and Granzyme B (GrzB) in AS patients were significantly reduced and negatively correlated with patient-reported disease activity. Thus, alteration in Th17 responses and the expression of cytotoxic-related molecules may contribute to the development of SpA.

Until now, the major focus on the contribution of IL-17 or cytotoxic factors (GrzA and GrzB) in the immunopathogenesis of SpA has been attributed to the CD4+ Th cells or CD8/NK cells, respectively (9, 12, 19, 20). Recently, also other cells like γδ cells and innate lymphoid cells were suggested to play a role in the disease pathogenesis (21, 22). IL-17 and cytotoxic molecules can also be produced by a subset of CD8+ T cells termed as mucosal-associated invariant T (MAIT) cells (23–26). These MAIT-cells are a type of innate-like CD8+ T cells and comprise up to 5% of the total T-cell population in peripheral blood and up to 20-40% of the T cell population in the liver (25, 27). MAIT-cells are characterized by the expression of a semi-invariant T cell receptor (TCR) α chain (Vα7.2-Jα33/12/20) and limited set of Vβ chains (28, 29). This invariant TCR recognizes the evolutionarily conserved MHC-like protein 1 (MR1) presenting riboflavin metabolites that can be synthesized by a wide range of bacteria and fungi (30–35). Upon TCR-dependent recognition of microbial antigen, MAIT cells display effector function by secreting pro-inflammatory cytokines (such as IL-17) and producing cytotoxic molecules (such as GrzA and GrzB) (36). Moreover, MAIT-cells share several markers with Th17 cells, such as CCR6 and the intracellular transcription factor retinoic acid-related orphan receptor γt (RORγt), and express a member of the killer lectin-like receptor CD161 which is widely expressed on the surface of CD8 T and NK cell (23, 24). In addition, MAIT-cells can upregulate KIR3DL2 on their surface so they can interact with HLA-B27 positive cells (8).

According to the aforementioned evidence, MAIT-cells may also contribute to the pathogenesis of SpA by producing the proinflammatory cytokine IL-17 or by secreting cytotoxic molecules.

Two studies reporting on MAIT-cells in the peripheral blood of AS patients demonstrated a decrease in circulating MAIT-cells in AS patients compared to healthy individuals, whereas an increase in MAIT-cells was observed in the synovial fluid of peripheral joint involvement in AS patients (16, 37). However, these studies included a broad phenotype of AS patients and included not only patients with primarily axial involvement, but also with peripheral joint involvement and patients who used biological (b)DMARD treatment. No sub-group analyses was done possibly due to lack of power. Therefore, conclusion about a direct association between the MAIT cells in the peripheral blood and axial involvement could not be made. As known, the pathogenesis in the axial involvement of spondyloarthritis may differ from peripheral disease (38).

Therefore, the objective of the present study was to quantify and characterize circulating MAIT-cells and their effector functions in a homogenous population of axSpA-patients defined with primarily axial involvement, the key manifestation in axSpA, without active peripheral involvement and/or extra-skeletal manifestations and/or current bDMARD treatment, and to compare these MAIT-cells and their function with age- and sex-matched healthy controls. Since MAIT cells have been identified to be impacted by immune-aging and age and CMV serostatus has been shown to reduce the number of circulating MAIT cells, we also aimed to explore if age and CMV status are associated with the presence of these MAIT cells in axSpA patients (39).

## Patients and methods

### Patients

Cross-sectional blood samples of consecutive patients who participated in the Groningen Leeuwarden Axial Spondyloarthritis (GLAS) cohort were obtained during a period of one year at a regular outpatient visit. Patients fulfilled the modified NY criteria for AS or the ASAS criteria for axial SpA. Patients with active peripheral arthritis and/or extra-skeletal manifestations such as inflammatory bowel disease, psoriasis or uveitis were excluded. Patients with active infections, current use of bDMARDs were also excluded.

Clinical data were available from the GLAS database including age, gender, HLA-B27 status, disease duration, smoking status, body-mass index (BMI) and medication use.

Furthermore, the AS Disease Activity Score (ASDAS) (40), Bath AS Disease Activity score (BASDAI, score 0-10) (41), and C-reactive protein (CRP, mg/L) were available as assessments for disease activity. Active disease was defined as ASDAS ≥ 2.1 or BASDAI ≥ 4.

The volunteer healthy controls (HC) were age and sex matched to the axSpA-patients using frequency matching of males and females of different age categories. The GLAS cohort was approved by the local ethics committees of the MCL and the UMCG (TPO364/604). All patients provided written informed consent according to the Declaration of Helsinki.

### Quantification of CD8^+^ T-cells and MAIT-cells

Absolute numbers of CD8^+^ T-cells were quantified by four-color FACS-Canto flow cytometer using TruCOUNT™ tubes (Becton&Dickinson) according to the manufacturer’s instructions. The absolute number of CD8^+^ T-cells was determined by comparing cellular events to beads events using CELL-Quest software (Becton&Dickinson). Absolute numbers of MAIT cells were calculated by multiplication of the percentages of MAIT cells within the CD8+ T cells by the absolute numbers of CD8+ T cells.

### Staining of cell surface antigens for flow cytometry

**Figure 1:**
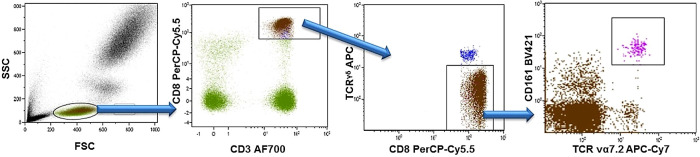
Gaitig strategy for CD8+ MAIT cells. Peripheral blood lymphocytes were identified and gated by their forward (FSC) and side (SSC) scatter. Next, CD3+CD8+ T cells were gated, *γδ* TCR+ cells were excluded, and MAIT cells were identified as TCRγδ-Vα7.2+CD161high cells.

Lithium-heparinized venous blood was obtained from patients and immediately after blood withdrawal, blood samples were aliquoted into 5 mL polypropylene tubes (Falcon^®^, Corning incorporated) at 100 uL per tube and stained with appropriate concentrations of the following fluorochrome-conjugated monoclonal antibodies: Alexa Fluor^®^ 700-conjugated anti-CD3 (from eBioscience, San Diego, CA, USA), *peridinin chlorophyll* protein complex with cyanin-5.5 (PerCP-CY5.5)-conjugated anti-CD8, allophycocyanin-C7 (APC-Cy7)-conjugated anti-TCRVα7.2, Brilliant Violet 605™ (BV605)-conjugated anti-CCR6 (all from BioLegend, San Diego, CA, USA), BV421-conjugated anti-CD161, allophycocyanin-C7 (APC)-conjugated anti-TCRγδ, fluorescein isothiocyanate (FITC)-conjugated anti-CD45RO, phycoerythrin-cyanin7 (PE-C7)-conjugated anti-CCR7 (all from BD Biosciences, Franklin Lakes, NJ, USA), and R-Phycoerythrin (*PE*)-*conjugated anti-KIR3DL2* (from *RM&D* Systems; Minneapolis, MN, USA). The appropriated isotype-matched control antibodies of irrelevant specificity were added to a separate tube as negative controls. Samples were incubated for 15 minutes at room temperature. Afterward, cells were treated with 2 mL diluted FACS lysing solution (BD Biosciences) for 10 minutes. Finally, the samples were washed in PBS containing 1% (w/v) bovine serum albumin (BSA), and immediately analysed on BD™ LSR II flow cytometer. Data were collected for 1 x 10^6^ events for each sample and plotted using Kaluza v1.2 (Beckman Coulter, Brea, CA, USA). Lymphocytes were gated for analysis based on forward and side scatter properties. Positively and negatively stained populations were calculated by quadrant dot-plot analysis, determined by the appropriate isotype controls. MAIT-cells were identified as TCRγδ^-^Vα7.2^+^CD161^high^ cells among the gated CD3^+^CD8^+^ T cells as described in **Figure 1**.

### Stimulation assay and immunofluorescence staining of intracellular proteins

Lithium-heparinized venous blood was obtained from all participants. Immediately after sampling, 200 μl blood was mixed with 200 μl RPMI1640 (Cambrex Bio Science, Verviers, Belgium), supplemented with 50 μg/ml gentamycin (Gibco, Paisley, Scotland, UK), and aliquoted into 5 ml polypropylene tubes (BD Biosciences, Amsterdam, The Netherlands)) (400 μl per tube). Diluted blood was stimulated *in vitro* with 5 ng/ml phorbol myristate acetate (PMA; Sigma-Aldrich) and 0.17 *µ*g/ml calcium ionophore A23187 (Ca-I; Sigma-Aldrich) for 16 h at 37°C and 5% CO_2_. As a negative control, 1 sample of each cell suspension remained without stimulation. Directly after addition of the stimulants, 10 *µ*g/ml brefeldin A (Sigma-Aldrich) was added. Brefeldin A was used to block intracellular transport mechanisms, thereby leading to an accumulation of cytokines in the cell. Culture tubes were incubated at 37°C, 5% CO_2_.

After stimulation, erythrocytes were lysed by adding 2.5 ml of ice cold amoniumchloride (pH 7.4). Samples were washed in wash buffer (PBS, 5% fetal bovine serum (FBS), 0.1% sodium azide (Merck, Darmstadt, Germany)) and the pellet was resuspended and stained with Alexa Fluor^®^ 700-conjugated anti-CD3, PerCP-CY5.5-conjugated anti-CD8, APC-Cy7-conjugated anti-TCRVα7.2, BV421-conjugated anti-CD161, APC-conjugated anti-TCRγδ, *PE*-c*onjugated anti-KIR3DL2*. Cells were fixed with 100 μl Reagent A (Caltag Laboratories, An der Grab, Austria) for 10 minutes. After washing, the pellet was resuspended in 100 μl permeabilization Reagent B (Caltag Laboratories, An der Grab, Austria) and labelled with Alexa Fluor 488-conjugated anti-IL17A and PE-CY7-conjugated anti-Granzyme-A (both purchased from eBioscience, San Diego, CA, USA), for 20 minutes in the dark. After staining, the cells were washed and immediately measured on an LSR-II flow cytometer (BD) and analysed with Kaluza Analysis Software (Beckman Coulter).

### Measurement of serum CMV IgG

Serum IgG specific for CMV was measured as described before (42). A 96-well ELISA plate (Greiner, Kremsmünster, Austria) was coated overnight with lysates of CMV-infected fibroblasts. Non-infected fibroblast lysates were used as negative controls. After coating, diluted serum samples were incubated. After 1 hour of incubation, goat anti-human IgG-HRP (Southern Biotech, Birmingham, AL, USA) was added and incubated for 1 hour. Next, samples were incubated with TBE substrate (Sigma-Aldrich, St. Louis, MO, USA) for 15 minutes and the reaction was stopped with sulfuric acid. The plates were scanned on a Versamax reader (Molecular Devices, Sunnyvale, CA, USA). A pool of sera from three CMV-seropositive individuals with known concentrations of CMV-specific IgG was used as reference to quantify levels of CMV-specific IgG in the tested samples. Values were reported in arbitrary units (AU/mL) (range 0-686) based on a standard serum pool. Values higher than 6 were considered seropositive.

### Statistical analysis

Statistical analysis was performed using GraphPad Prism (version 7.0) or SPSS version 25.0 (IBM). Normal distribution of data was assessed. Differences in numbers and percentages of cells between axSpA-patients and HC were compared using Mann-Whitney U test corrected with Dunn’s test in case of multiple testing. *P*-values <0.05 were considered statistically significant. Associations of MAIT-cells with age and CMV titres were explored using Spearman’s correlation coefficient and interpreted as poor association (0.0-0.2), fair (0.2-0.4), moderate (0.4-0.6) good (0.6-0.8) or excellent (0.8-1.0) (43).

## Results

### Patient characteristics

In total, 41 axSpA-patients and 30 age and sex matched HC were included. Median age of the axSpA-patients was 49.9 (IQR 28.8-54.8) years, 73% were male. The axSpA-patients had a median ASDAS of 2.6 (1.9-3.4) and median BASDAI of 4.8 (2.4-6.9). Of the 41 axSpA-patients, 28 (70%) had active disease according to ASDAS (≥2.1) and 22 (54%) according to BASDAI (≥4). Only 3 (7.3%) of the axSpA-patients used a bDMARD (TNF-α blocking agent) previously, which was discontinued at least 6 months before inclusion in this study. Only 2 (4.8%) patients reported a history of extra-skeletal manifestation (1 IBD and 1 psoriasis) which were both not active at time of inclusion. Positive CMV status was significantly different between axSpA-patients and HC, 59% versus 33% respectively. Only age was significantly different between axSpA-patients with CMV+ and CMV- status. All characteristics of axSpA-patients and HC are depicted in **Table 1**.

**Table 1:** Characteristics of axSpA-patients and age- and sex-matched HC.

	Axial SpA patients (n=41)	Healthy controls (n=30)
Age	49.9 (28.8-54.8)	49.5 (29.0-57.3)
Male gender	30 (73.2)	22 (73.3)
CMV +	24 (58.5) *	10 (33.3)
HLA-B27+	32 (78.0)	–
Symptom duration	22.6 (10.9-31.4)	–
Time since diagnosis	11.1 (4.5-20.6)	–
Current NSAID use	31 (75.6)	–
Previous DMARD use	1 (2.4)	–
Precious anti-TNF use	3 (7.3)	–
BASDAI	4.8 (2.4-6.9)	–
ASDAS	2.6 (1.9-3.4)	–
CRP	3.3 (2.0-7.0)	–

Data presented as median (IQR), or number (percentage); statistics preformed using Mann-Whitney-U test. *p < 0.05.

### No difference in numbers and frequencies of circulating MAIT-cells between axSpA-patients and HC

**Figure 2:**
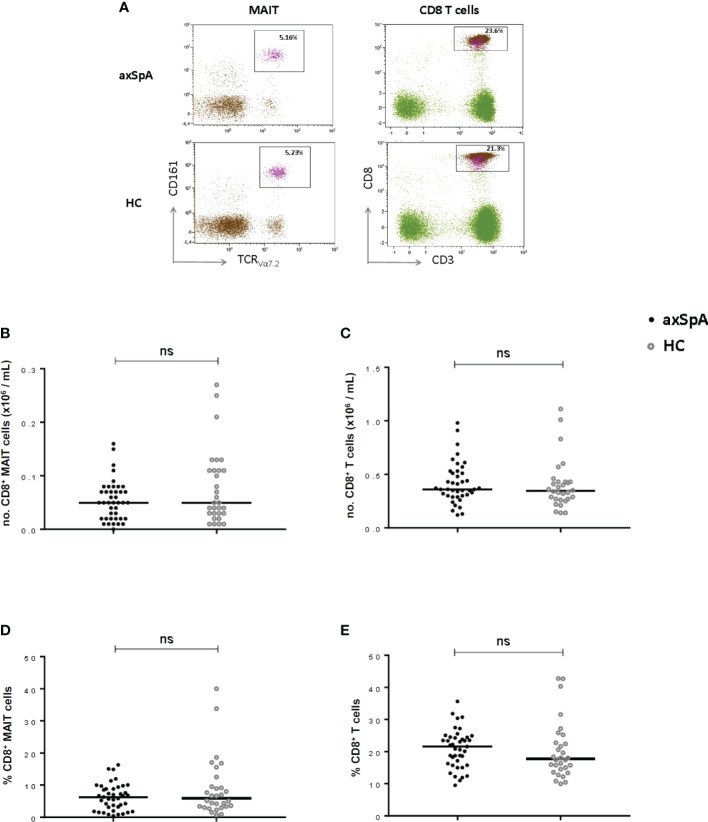
MAIT cells and CD8+ T cells in peripheral blood of axial SpA patients and healthy controls. Representative flow cytometry dot plot graphs showing the percentage of CD8+ MAIT cells (left plots) and CD8+ cells (right plots) in axSpA-patient (upper line) and in HC (lower line) **(A)**. Numbers and percentages of MAIT-cells **(B, D)** and CD8+T cells **(C, E)** were determined by flowcytometry in peripheral blood of 41 axial SpA patients (axSpA) and 30 matched HCs. Data presented as median or percentage; statistics preformed using Mann-Whitney-U corrected with Dunn’s test. ns, non-significant.

To explore the possible involvement of MAIT-cells in the disease process of axSpA, we compared numbers and percentages of circulating MAIT-cells between axSpA-patients and age and sex matched HC (**Figure 2A**). No significant differences were observed in both numbers and percentages between axSpA-patients and HC (**Figures 2B, D**). Since MAIT-cells were determined among the CD8+ T cell population, we assessed the differences in numbers and percentages of circulating CD8+ T cells between axSpA and HC. As shown in **Figures 2C, E**, CD8+ T cells did not show any significant differences in numbers and percentages.

### Decreased number of circulating central memory MAIT-cells in axSpA-patients

**Figure 3:**
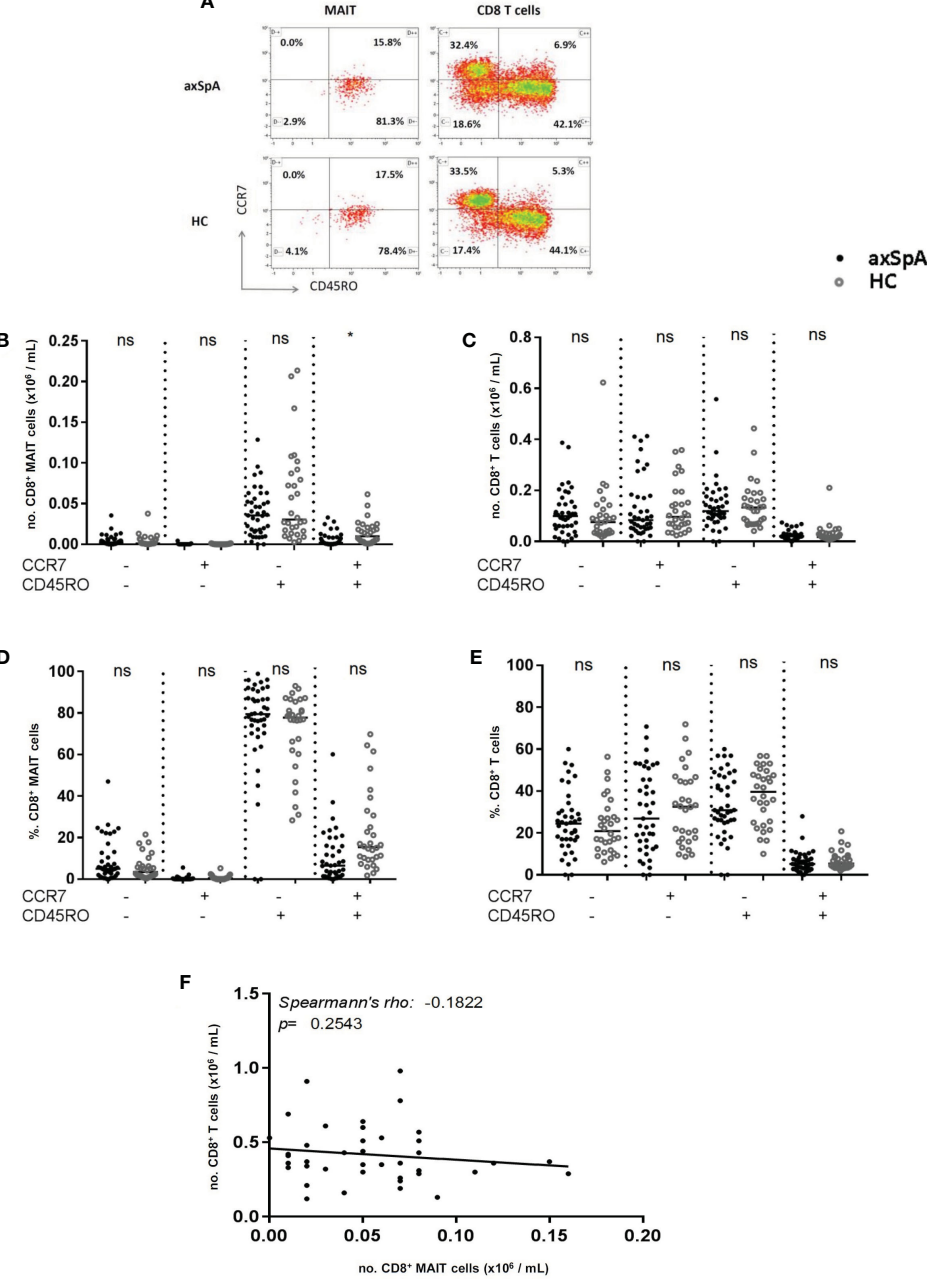
Distribution of CD8+ MAIT cell subsets and CD8+ T cell subsets in peripheral blood of axial SpA patients and healthy controls. Representative flow cytometry dot plot analyses of peripheral blood CD8+ MAIT cell subsets (left plot) and CD8+ T cells subsets (right plot) assessing surface expression of CCR7 and CD45RO in axSpA patient (upper plots) and HC (lowe plots) **(A)**. Numbers and percentages of MAIT cell subpopulations **(B, D)** and CD8+ T cell subpopulations **(C, E)** were determined by flowcytometry in peripheral blood of 41 axial SpA patients (axSpA) and 30 matched HCs. Subsets from circulating MAIT-cells and CD8+ T cells were identified based on the expression of CCR7 and CD45RO and subclassified into naïve (CD45RO-CCR7+), central memory (CD45RO+CCR7+), effector memory (CD45RO+CCR7) and terminal differentiated (CD45RO-CCR7-) subpopulations. Correlation between numbers of MAIT-cells and numbers of CD8+ T cells in peripheral blood of axSpA-patients **(F)**. Data presented as median x10^9 cells/L or (%) (IQR); statistics performed using Mann Whitney-U corrected with Dunn’s test. Correlations were assessed using Spearman’s correlation coefficient. ns, non-significant; *p<0.05.

The distribution of the different subtypes of MAIT-cells was assessed according to their surface markers. CCR7 and CD45RO were stained to characterize the MAIT-cells into different subtypes, namely naïve (CD45RO-CCR7+), central memory (CD45RO+CCR7+), effector memory (CD45RO+CCR7-) and terminally differentiated (CD45RO-CCR7-) cells (**Figure 3A**). The number of central memory MAIT-cells was significantly decreased in axSpA-patients compared to HC (**Figure 3B**). However, the percentage of central memory MAIT-cells did not significantly differ between patients and HC (p=0.11) (**Figure 3D**). In addition, no significant differences were observed in numbers and percentages of naïve, effector memory and terminally differentiated MAIT-cells in axSpA-patients and HC.

We next assessed whether the decrease in central memory MAIT-cells was attributed to a decrease in CD8+ central memory T cells. No significant difference was observed in the numbers of CD8+ central memory T cells between axSpA-patients and HC (**Figure 3C**). Also, the numbers of naïve, effector memory and terminally differentiated CD8+ T cells did not differ. The same results were found for the percentages of CD8+ T cell subsets (**Figure 3E**). Also, no correlation was observed between MAIT cell numbers and CD8+ T cells numbers in peripheral blood of axSpA-patients (**Figure 3F**). Collectively, the number of circulating central memory MAIT cells was significantly reduced in axSpA-patients compared to HC, whereas no significant difference was found in the distribution of other MAIT cell subsets between both groups. The decrease in central memory MAIT cells of axSpA-patients was not attributed to the decrease in CD8+ T cells.

### Circulating MAIT-cells of axSpA patient have a decreased cytotoxic potential

**Figure 4:**
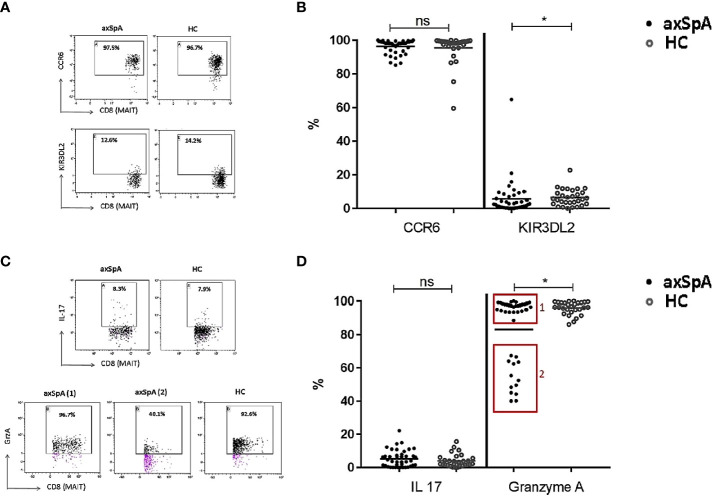
Pro-inflammatory and cytotoxic potential of CD8+ MAIT cells in peripheral blood of axial SpA patients and healthy controls. Representative flow cytometry dot plot graphs **(A)** showing the expression percentage of CCR6 (upper line) and KIR3DL2 (lower line) on CD8+ MAIT cells in axSpA-patient (left plots) and in HC (right plots), and the representative dot plots **(C)** showing the percentage of CD8+ MAIT cells producing IL-17 (upper plots) and GrzA (lower plots) in axSpA-patient (left plots) and in HC (right plots). Percentage expression of surface CCR6 and KIR3DL2 **(B)** and intracellular IL-17 and Granzyme A **(D)** in CD8+ MAIT-cells from 41 axSpA-patients and 30 matched HCs were measured by flowcytometry. Data presented as individual values with median percentages; statistics preformed using Mann-Whitney-U corrected with Dunn’s test. ns, non-significant; *p<0.05.

To determine the pro-inflammatory potential of MAIT-cells, we assessed their surface expression of CCR6 and KIR3DL2, which have been shown to be enriched for production of the pro-inflammatory cytokine IL-17. As shown in **Figure 4**, no differences were observed in the percentage of CCR6 expressing cells, whereas a significant decrease were found in the expression of KIR3DL2 on MAIT-cells from axSpA-patients compared to those from HCs. There were no statistically significant differences in CCR6 and KIR3DL2 mean fluorescence intensity (MFI) levels on MAIT cells of axSpA patients compared to HCs, suggesting a reduction of the KIR3DL2+ population of MAIT cells rather than decreased expression per cell (**Supplementary Figure 1**).

We next evaluated the production of IL-17 by MAIT-cells upon *in vitro* stimulation as described in the method section. Expression of IL-17 in MAIT-cells of axSpA-patients was comparable to that of HC.

To determine the cytotoxic potential of MAIT-cells, intracellular production of Granzyme A was also evaluated following *in vitro* activation. Granzyme A was selected according to it higher level of production by CD8+ T cells compared to Granzyme B (44). Interestingly, Granzyme A producing MAIT-cells in axSpA patient were significantly reduced in comparison to that of HC (**Figure 4D**). Notably, it seemed two groups could be distinguished: group 1 consisting of 28 patients with nearly comparable Granzyme A production to HC and group 2 consisting of 13 axSpA patients with a lower Granzyme A production. Clinically, the patients in group 2 seem to have a higher disease activity (ASDAS 3.0 (1.9-3.4), BASDAI 5.0 (2.1-7.1)) compared to group 1 (ASDAS 2.5 (1.9-3.6), BASDAI 3.5 (2.4-6.4)). The numbers of patients in these groups were too small to reach statistical significance. MAIT-cells of axSpA-patients seem to have a reduced cytotoxic potential as compared with those of healthy individuals.

### Age and circulating MAIT-cells in axSpA-patients

The age-related changes in immune functions, called immunosenescence, are the cause of the increased susceptibility to infections and inflammatory diseases in elderly. Therefore, we explored the effect of age on the numbers of MAIT-cells in axSpA-patients, especially because axSpA starts at a relatively early age. As shown in **Table 2**, age was inversely correlated with the total number of MAIT-cells in axSpA patients (rho= -0.312; p=0.047) and HC (rho= -0.2913, p 0.065). However, no correlations were observed between age and different MAIT cell subsets (**Table 2**). Age was also not correlated with the percentages of IL-17 and Granzyme A positive MAIT-cells (**Table 2**).

**Table 2:** Correlation of MAIT cells with age and CMV titre.

Age				
Total rho= -0.312 ***p=0.047***	**CCR7-CD45RO-**	**CCR7+CD45RO-**	**CCR7-CD45RO+**	**CCR7+CD45RO+**
	rho= -0.197 *p= 0.217*	rho= -0.303 *p=0.054*	rho= 0.293 *p= 0.063*	rho= -0.152 *p=0.343*
	**CCR6+**	**KIR3DL2+**	**IL17+**	**GrzA+**
	rho= -0.057 *p=0.726*	rho= 0.040 *p=0.803*	rho= -0.019 *p=0.903*	rho= 0.219 *p=0.168*

CMV titer				
Total rho= -0381 ***p=0.017***	CCR7-CD45RO-	CCR7+CD45RO-	CCR7-CD45RO+	CCR7+CD45RO+
	rho= 0.040 *p=0.809*	rho= -0.226 *p=0.166*	rho= 0.284 *p=0.080*	rho= -0.331 ***p=0.039***
	CCR6+	KIR3DL2+	IL17+	GrzA+
	rho= -0.090 *p=0.587*	rho= -0.045 *p=0.787*	rho= 0.167 *p=0.309*	rho= 0.067 *p=0.690*

Correlations of age and CMV titers with numbers of circulating MAIT cell subsets, percentages of MAIT cells expressing CCR6 or KIR3DL2, and percentages of MAIT cells producing IL-17 or GrzA. The numbers represent the Spearman’s correlation coefficient for each correlation. Significant correlations have a p-value highlighted in bold.

### CMV serostatus and circulating MAIT-cells

The rate of CMV infections increases with age and has been suggested that it exacerbate immunosenescence which may impact the processes of T cell homeostasis and differentiation. Indeed, CMV serostatus has been shown to impact CD161 expressing T-cells with a numerical decline (39, 45). Furthermore, an increased mortality has been seen associated with CMV antibody titres (46, 47). Therefore, we explored the effect of CMV infection on the presence of circulating MAIT cells in axSpA patients. Patients were stratified based on CMV IgG status, where a serum IgG titer >6 AU/mL was considered seropositive (**Table 1**). No differences in numbers or percentages of MAIT-cells and MAIT cell subsets were observed between the CMV positive and negative individuals (data not shown). As shown in **Table 2**, serum CMV-IgG titers correlated inversely with the total number of circulating MAIT-cells of patients (rho= -0.381; p=0.017). This correlation was as expected also found in HC (rho -0.399, p=0.029). Additionally, a significant inverse correlation was observed beteween numbers of central memory MAIT-cells and CMV-IgG titers in axSpA-patients (**Table 2**). No correlation between cytokine production and CMV titre was found.

## Discussion

The present study provides unique and original data on MAIT-cells in axSpA-patients with primarily axial disease without active peripheral arthritis and/or extra skeletal manifestations and/or current bDMARD treatment. In this study, we demonstrated that numbers and percentages of circulating MAIT-cells in axSpA-patients were similar to those in the HC group. However, a decreased number of a MAIT-cells subset, expressing the phenotype of central memory, was observed in axSpA-patients. The expression level of IL-17 in MAIT-cells from axSpA-patients and HC were comparable. Interestingly, circulating MAIT-cells from axSpA-patients seem to have a reduced cytotoxic potential which is reflected by a reduction in Granzyme A production. The decrease in Granzyme A producing MAIT-cells was more obvious in patients with higher disease activity.

A previous study by Gracey et al. reported a reduction in the frequency of MAIT-cells in peripheral blood of AS patients, which is inconsistent with our results (16). This reduction might be explained by the differences in the patient characteristics. We included AS patient with primarily axial involvement. Furthermore, our patients were older (mean 49.9 versus 40 years) and consequently had overall longer disease duration and perhaps most important, our patients did not use any bDMARD whereas 61% of the patients in the study by Gracey et al. used TNF blocking therapy with a mean BASDAI of 4.3. The use of bDMARD, primarily TNF blocking therapy, might intervene with the signalling pathways related to the inflammatory response in axSpA. Therefore the use of bDMARDs might lead to a different response from circulating MAIT cells and might lead to different frequencies or phenotypes of circulating MAIT cells

Also, a study of Hayashi et al. found a significant decrease in the number of MAIT-cells in the peripheral blood of patients diagnosed with AS according to the modified NY criteria (37). Their population of AS patients was also significantly younger than our patients (median age 34.5 versus 49.9 years) and had less active disease (median ASDAS 2.3 versus 2.6, BASDAI 3.3 versus 4.8). Furthermore, half of their patients used TNF blocking therapy Toussint etal included AS patients fulfilling the modified NY criteria without bDMARD use and reported also decrease in circulating MAIT cells (48). However, almost half of the patients had extra-skeletal manifestations which may have influenced the results.

In respect to the subtypes of MAIT-cells, we found a significant decrease in the number of circulating central memory MAIT-cells in axSpA-patients compared to HC. Previous studies did not define the different subtypes of MAIT-cells. It is unclear if the decrease in central memory MAIT-cells is due to enhanced migration to the secondary lymphoid organs or due to enhanced development towards effector memory MAIT-cells. Further research is required to clarify the decrease in these circulating central memory MAIT-cells in axSpA-patients.

In axSpA no immunological auto-antigens have been identified. MAIT cells however express a semi-invariant TCR which recognizes the conserved MHC-like protein 1 (MR1). The MR1 ligand binds to vitamin-B-based antigens and can present these in axSpA patients, possibly originating in the gastro-intestinal tract after infection. This process is hypothesized to start the auto-immune cascade leading to axSpA (35, 49). Furthermore, it seems that the pathogenesis of axSpA is more cytokine driven. IL-17 has been identified as an important factor in this immunopathogenesis (10, 11). We hypothesized that MAIT-cells might contribute to the immunopathogenesis of axSpA by producing IL-17. Our study, however, found no significant differences in IL-17 production by MAIT-cells between axSpA-patients and HC. Interestingly, we did observe a reduction in the frequency of Granzyme A producing MAIT-cells in peripheral blood of axSpA-patients in comparison to HC. In line with our data, Gracey and co-workers have previously demonstrated a down-regulation of cytotoxic cell-related genes in whole blood from AS patients (50). Consistent with these findings, Ren et al. have shown that Granzyme A level was significantly decreased in serum of AS-patients (18). They hypothesized that reduction in circulating cytotoxic cells in AS-patients is caused by recruitment of these cytotoxic cells to the inflamed joint and implicated in tissue injury. Accordingly, decreased Granzyme A-producing MAIT-cells in peripheral blood of axSpA-patients might also be explained by their migration and accumulation at the site of inflammation. Indeed, enrichment of MAIT-cells in synovial fluid of AS-patients was confirmed by Gracey and colleagues (16). Furthermore, Santiago etal investigated mouse models of Granzyme A deficient mouse and found Granzyme A to be contributory to osteoclast differentiation. Granzyme A may also have influence on the RANK-L pathway and anti-TNF production and in this way intervene in the bone remodelling process in axSpA (51). Further studies are warranted to further explore the pathogenic role of MAIT-cells in axSpA-patients including the effect of disease activity on Granzyme A production by MAIT cells.

Circulating MAIT-cells of AS-patients have shown to decrease with age (39, 52–54). In addition to the impact of age, CMV status were also shown to be associated with accelerated immunosenescence (55). Indeed, reduced numbers of circulting MAIT cells have been reported in CMV-seropositive donors compared to CMV-seronegative donors (45). Therefore, we assessed the impact of age and CMV status on circulating MAIT cells in our study population. Total numbers of MAIT cells in axSpA-patients were negatively correlated with both age and CMV-titer. This is in concordance with other data provided by van der Geest et al. who described a negative association between age and numbers of circulating CD161^high+^TCR V_α7.2_^+^CD8^+^ T cells (MAIT-cells) in healthy subjects (39). Analysing the different subsets of MAIT-cells in axSpA-patients, we found that CMV-IgG titer was negatively correlated with central memory MAIT-cells however no specific significant associations were observed with age. This may indicate the involvement of CMV in aberrant distribution of MAIT-cell subsets. Although the pathogenesis of SpA has yet to be fully elucidated, the role of MAIT-cells in mucosal immunity might suggest that these cells are potential players in SpA. Further research is needed to examine the antigen-specificity of memory MAIT-cells in SpA-patients.

## Conclusion

Our cross-sectional study of axSpA-patients with primarily axial disease, without active peripheral arthritis and/or extra-articular manifestations and/or current bDMARD treatment, showed no significant differences in numbers and percentages of circulating MAIT-cells compared to age- and sex-matched HC. These MAIT-cells produces the same amount of IL-17 as in those of HCs. However, when analysing the distribution of different MAIT cell subtypes, we demonstrated that numbers of central memory MAIT-cells, as well as the Granzyme A producing MAIT-cells were significantly decreased in peripheral blood of axSpA-patients in comparison to HC. Numbers of circulating central memory MAIT-cells appears to be related to the strength of the immune response against CMV, as they correlate negatively with the CMV-IgG titer. The decrease in MAIT-cells with cytotoxic capability in the peripheral blood of this homogenous group of axSpA-patients with primarily axial involvement might implicate that these cell types migrate to the inflamed tissue at axial sites and are involved in the axial disease pathogenesis. Further research is necessary to examine the phenotype and the distribution of MAIT-cells at the site of inflammation. Also longitudinal studies assessing numbers and cytotoxic function of MAIT cells at various stages of disease, including subgroups with different extra-skeletal manifestations, are warranted.

## Data availability statement

The original contributions presented in the study are included in the article/**Supplementary Material**. Further inquiries can be directed to the corresponding author.

## Author contributions

All authors listed have made a substantial, direct, and intellectual contribution to the work, and approved it for publication.
