# Supplementary figures and images for: Mucosal-associated invariant T cells in patients with axial spondyloarthritis
Source: Front Immunol. 2023 Mar 10;14:1128270. doi: 10.3389/fimmu.2023.1128270 (PMC10038212; doi:10.3389/fimmu.2023.1128270)

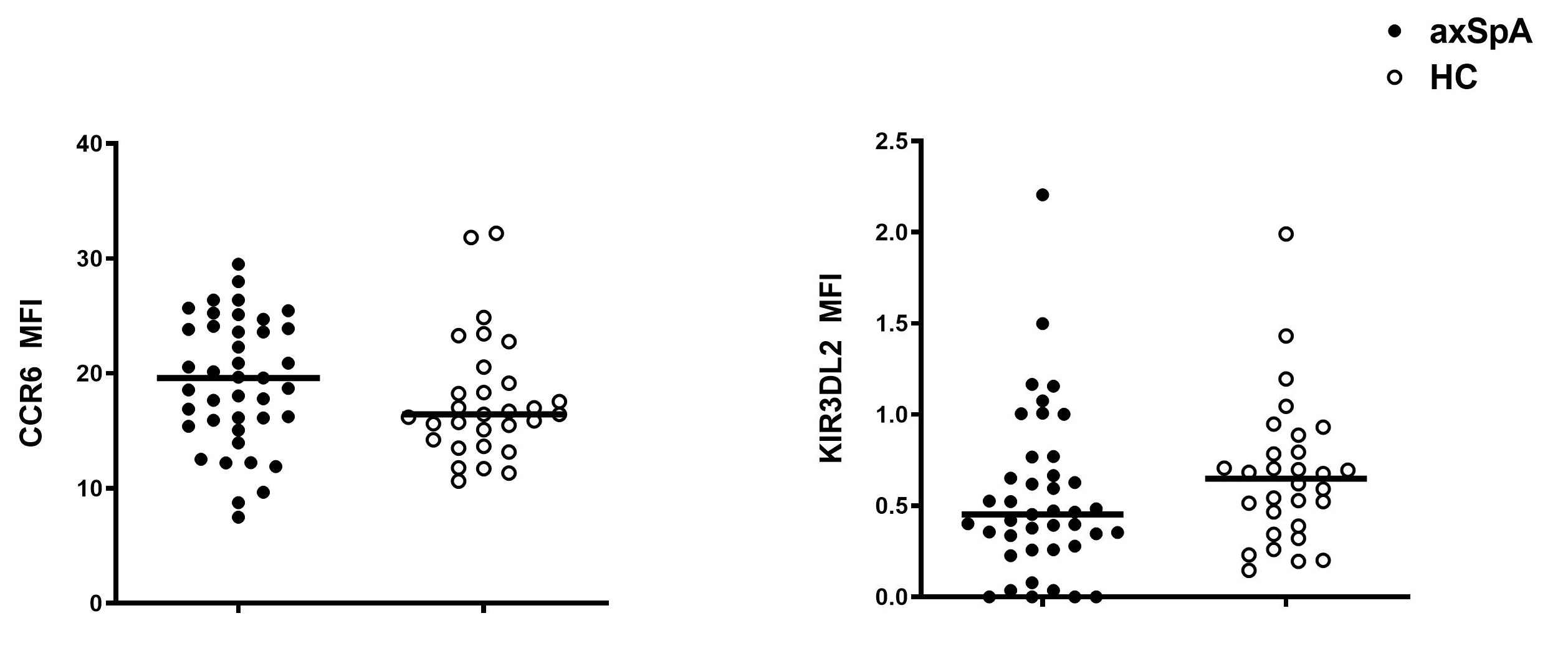

Supplement: Supplementary file 1 [file Image_1.jpeg]
